# Lignin Nanoparticles from Coffee Grounds Enhance the Cytotoxicity of Coffee Grounds Lignin and Induce Apoptosis in Colorectal Cancer (CRC) Cell Lines

**DOI:** 10.3390/nano15231754

**Published:** 2025-11-22

**Authors:** Shiwen Liu, Xin Zhang, Jihui Wang, Xuan Chen, Xiaoqin Pan, Junqiu Zhang, Jingyu Xu, Weibin Bai

**Affiliations:** 1 Department of Food Science and Engineering, Institute of Food Safety and Nutrition, Guangdong Engineering Technology Center of Food Safety Molecular Rapid Detection, Jinan University, Guangzhou 510632, China; 2 College of Materials Science and Engineering, Qiqihar University, Qiqihar 161006, China; 3 School of Life and Health Technology, Dongguan University of Technology, Dongguan 523808, China

**Keywords:** lignin nanoparticles, coffee grounds, colorectal cancer

## Abstract

Colorectal cancer (CRC) is the third most common malignant tumor worldwide, with morbidity and mortality rates increasing annually. Consequently, the development of safe and effective natural anti-tumor drugs has become a critical research focus. Lignin, a polyphenolic polymer abundant in nature, possesses a unique chemical structure that imparts antioxidant, anti-inflammatory, and tumor cell-related bioactivities, demonstrating remarkable potential in the prevention and treatment of CRC. In this study, we investigated the anti-tumor cell effects of coffee grounds lignin nanoparticles (CLN), prepared using aqueous ethanol extraction from coffee waste, against three CRC cell lines (HCT116, HT29, SW620). The results indicated that CLN potently inhibited the proliferation of all three CRC cell lines mentioned above. This research supports the transformation of lignin from natural products into innovative anti-colorectal cancer agent, offering new insights for the development of novel therapeutic strategies characterized by low toxicity and high efficacy. As research advances and technology continues to innovate, lignin-based nanoparticles emerge as key natural products in CRC therapy, offering new hope for patients and paving the way for the application of natural products in cancer therapy.

## 1. Introduction

Colorectal cancer is one of the major diseases that poses a significant threat to human health worldwide [1,2]. According to data released by the International Agency for Research on Cancer (IARC) of the World Health Organization in 2024, there are projected to be over 2 million new cases of colorectal cancer and approximately 1 million deaths globally each year [3]. While traditional treatments such as surgical resection, chemotherapy, and radiotherapy can extend patient survival to some extent, they are associated with challenges such as a high postoperative recurrence rate, chemotherapy resistance, and significant side effects from radiotherapy. These issues severely impact the quality of life and prognosis for patients [4,5].

Natural products have emerged as a prominent focus in CRC therapy research due to their diverse origins, favorable safety profiles, and multifaceted mechanisms of action [6,7,8,9,10]. Lignin, the second most abundant natural organic polymer after cellulose, is widely present in plant cell walls, with a global biosynthesis estimated at up to hundreds of billions of tons per year [11]. Historically, lignin has been incinerated as a by-product of the paper industry, lingin’s disposal has contributed to resource waste and environmental pollution [11]. However, recent in-depth research on lignin has gradually unveiled its antioxidant, anti-inflammatory, and antibacterial properties, attributed to its rich array of active groups [11,12]. Consequently, the potential applications of lignin in the field of CRC therapy have become increasingly significant [13,14].

It has been established that the development of CRC is closely associated with oxidative stress, inflammatory responses, intestinal microecological imbalances, and the activation of abnormal cell signaling pathways [15,16,17,18,19]. The multi-targeting nature of lignin in CRC therapy effectively intervenes in the critical processes involved in the development of CRC. Its unique chemical structure and biological activity suggest that lignin may offer a novel strategy for treating CRC by regulating the intestinal microenvironment, inhibiting tumor cell proliferation, inducing apoptosis, and engaging other therapeutic pathways [14]. Therefore, exploring the potential applications of lignin in the treatment of CRC is expected to not only foster innovative therapeutic approaches but also provide a direction for the high-value utilization of lignin. This research holds significant theoretical importance and clinical value.

Based on the above background, in this experiment, lignin nanoparticles were prepared from coffee grounds, referred to as coffee lignin nanoparticles (CLN), using the aqueous ethanol extraction method, and the inhibitory effects of these CLN on CRC cell lines (HCT116, HT29, SW620) were evaluated. This study not only facilitates the high-value valorization of coffee grounds waste but also anticipates representing a significant breakthrough in the field of natural antitumor drugs. Its importance extends beyond the treatment of a single disease, embodying a comprehensive innovative approach encapsulated in the concept of “waste valorization—active ingredient—functional drug”. This research provides a replicable model for the synergistic development of the pharmaceutical and environmental protection industries.

## 2. Experimental Section

### 2.1. Material

Coffee grounds were provided from coffee beans purchased from Yunnan Zhongfei Food Co., Ltd. (Kunming, China). Dulbecco’s modified Eagle’s medium (DMEM), phosphate-buffered saline (PBS), the CCK8 kit, and the mitochondrial membrane potential assay kit with JC-1 were purchased from Meilunbio Co., Ltd. Dalian, China. Fetal bovine serum (FBS) was purchased from HyClone Laboratories (Logan, UT, USA). All cell lines used in this study were obtained from the Chinese Academy of Sciences (Beijing, China). The other reagents mentioned in the article were purchased from Shanghai Aladdin Biochemical Science and Technology Co., Ltd. (Shanghai, China), and were used as received without further purification.

### 2.2. Preparation

The air-dried coffee grounds are crushed using a pulverizer (BenChen, Shijiazhuang, China) and then passed through a 60-mesh sieve to obtain coffee grounds powder. Weigh 100 g of the powder coffee grounds, place it in a vacuum drying oven (KLYQ, Shijiazhuang, China), and dry at 60 °C until a constant weight is achieved. Set aside for later use.

Weighing 50 g of pre-treated powder coffee grounds, it was placed in a cooking jar, and a 60% aqueous ethanol solution was added at a ratio of 1:10 (*w*/*v*, g/mL). The cooking jar was then placed in a steam cooker and extracted by heating at 175 °C for 150 min. After extraction, the solution was cooled to room temperature, transferred to a centrifuge tube, and centrifuged at 4000 r/min for 10 min to collect the supernatant. The supernatant was poured into a beaker, and hydrochloric acid was added slowly while stirring to adjust the pH to 2, facilitating the precipitation of lignin. After standing for 2 h, the precipitate was collected by filtration using a filtration device. The precipitate was washed with deionized water until neutral, then placed in a vacuum drying oven and dried at 60 °C until a constant weight was achieved to obtain crude lignin. The crude lignin was dissolved in an appropriate amount of ethanol and purified by column chromatography, using anhydrous ethanol as the eluent (Sephadex LH-20 serves as stationary phase). The eluent was collected, concentrated by rotary evaporation, and then vacuum dried to obtain purified lignin (CL).

2 g of lignin was added to 100 mL of tetrahydrofuran and stirred ultrasonically for 2 h to ensure complete dissolution. Using a peristaltic pump, 120 mL of deionized water was added to the solution at a rate of 3.75 mL/min. The mixture was centrifuged. The resulting precipitate was rinsed five times with deionized water and dried in an oven at 60 °C for 24 h. The preparation of coffee lignin nanoparticles (CLN) was completed.

### 2.3. Characterization of Coffee Lignin (CL) and Coffee Lignin Nanoparticles (CLN)

2D HSQC NMR results were recorded on a Bruker Avance III HD 400 MHz instrument with a BBFO probe equipped with a Z-gradient coil for structural analysis. Data were processed with MestreNova (Mestrelab Research) software (MestReNova 16 for Windows) using a 90° shifted square sine-bell apodization, window, baseline and phase correction were applied in both directions.

The morphology of the samples was observed by SEM (JSM-7800F, Shimadzu, Japan).

### 2.4. Determination of Cell Viability

The cytotoxicity of coffee lignin (CL) and coffee lignin nanoparticles (CLN) on the human colon cell line NCM460, as well as on colorectal cancer (CRC) cell lines SW620, HT29, and HCT116 were analyzed using the CCK-8 assay. Cells (NCM460, SW620, HT29, or HCT116) were harvested during their logarithmic growth phase and subsequently plated in 96-well plates. Cells were seeded in 96-well plates at a density of 5 × 10^3^ cells per well in 100 μL of DMEM (*n* = 3). Following a 24 h incubation period, varying concentrations of CL or CLN (50, 100, 200, 400, and 800 μg/mL) were introduced to each well. Concurrently, untreated control wells (containing cells and medium with 0.8% DMSO only) and blank control wells (without cells) were established for comparative analysis. In particular, CL and CLN were first dissolved in a small volume of DMSO to prepare a high-concentration stock solution. This stock solution was then dispersed and diluted in the DMEM to obtain the working concentrations used in the treatments. The final concentration of DMSO in the culture medium never exceeded 0.8% (*v*/*v*). This protocol was applied consistently throughout the study unless otherwise specified. Following a 24 h incubation period, varying concentrations of CL or CLN (50, 100, 200, 400, and 800 μg/mL) were introduced to each well. Concurrently, untreated control wells (containing cells and medium only) and blank control wells (without cells) were established for comparative analysis. Following a 24 h incubation period with the specified pharmacological agents, 10 μL of the kit reagent was added to 100 μL of cell monolayers and incubated for an additional 60 min at 37 °C. Subsequently, the optical densities were determined using an enzyme-linked immunosorbent assay (ELISA) reader at a wavelength of 450 nm. The cell viability rate, expressed as a percentage, was determined using the following formula:

(OD ^a^ experimental group − OD blank control)/(OD control group − OD blank control) × 100%

The cell inhibition rate, expressed as a percentage, was calculated using the following formula:

(OD control group − OD experimental group)/(OD control group − OD blank control) × 100%

^a^ OD = optical density

### 2.5. Determination of Clone Formation Ability

The CRC cell lines (SW620, HT29, and HCT116) were seeded in 6-well plates at densities of 500 cells per well in 2 mL of DMEM, respectively (*n* = 3 per group), and incubated at 37 °C with 5% CO_2_ for 24 h. Following this initial incubation, cells were treated with varying concentrations of CLN (50 μg/mL, 100 μg/mL, 200 μg/mL) and maintained in culture for a period of two weeks. The cell culture medium was refreshed every three days. After the two-week incubation period, cells were washed twice with phosphate-buffered saline (PBS) and fixed with paraformaldehyde for 30 min. Subsequently, cells were stained with a 0.1% solution of crystal violet for 30 min. Following staining, cells were air-dried overnight. The intensity of crystal violet staining was quantified via ImageJ software (version 2.0.0).

### 2.6. Scratch Assay

CRC cells were seeded in 6-well plates at a density of 2 × 10^5^ per well in 2 mL of DMEM, and cultured under standard conditions (37 °C, 5% CO_2_) until they reached approximately 90% confluence. A standardized linear scratch was created using a sterile pipette tip. Subsequently, the cells were treated with various concentrations of CLN (50 μg/mL, 100 μg/mL, 200 μg/mL) for 48 h. To evaluate migratory capacity, images were captured at 0, 24, and 48 h post-scratch using an inverted fluorescence microscope (Axio Observer 7, Carl Zeiss, Oberkochen, Germany). The extent of cell migration was quantified via ImageJ software (version 2.0.0), enabling comparative analysis of CLN’s on CRC cells motility.

### 2.7. Detection of Mitochondrial Membrane Potential Damage Using a Inverted Fluorescent Microscope

CRC cells were seeded at a density of 2.5 × 10^5^ cells in 2 mL of DMEM per well in 6-well plates (*n* = 3 per group) and treated with varying concentrations of CLN (50 μg/mL, 100 μg/mL, 200 μg/mL) at 37 °C with 5% CO_2_ for 24 h. Subsequently, mitochondrial membrane potential was evaluated using the JC-1 kit. Immunofluorescence images were acquired using an inverted fluorescence microscope (Axio Observer 7, Carl Zeiss, Oberkochen, Germany).

## 3. Results and Discussion

**Figure 1:**
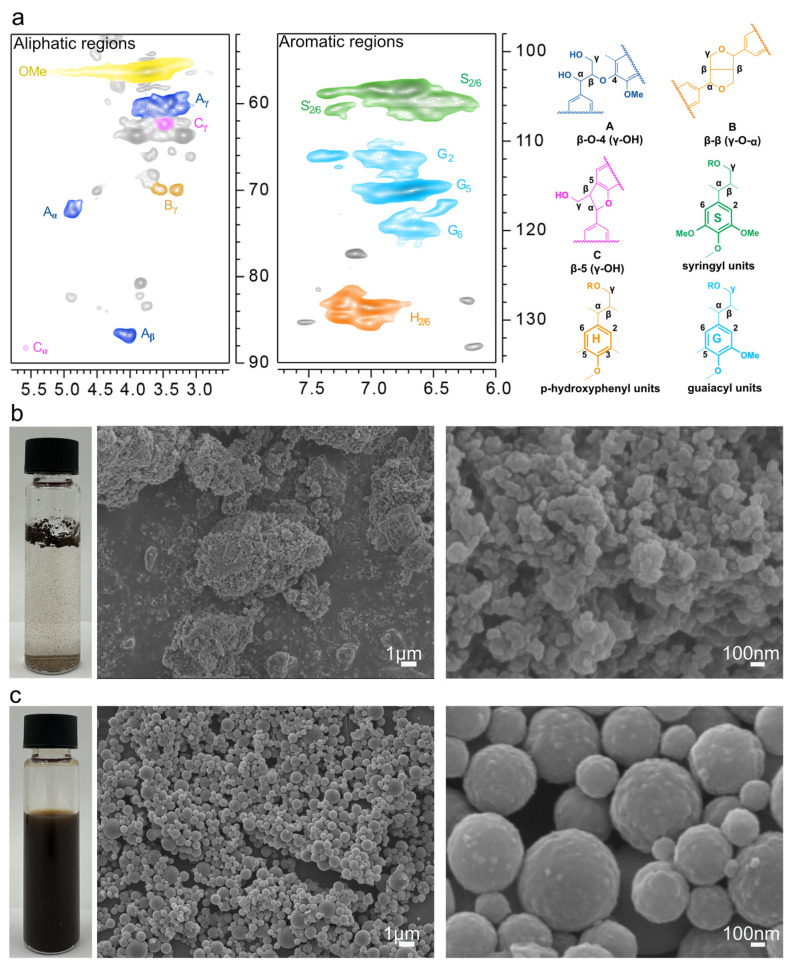
(**a**) 2D-HSQC NMR spectra of lignin samples (lignin structure), (**b**) on the left: dispersion of pristine lignin in PBS buffer solution (pH 7.4), on the right: microscopic morphology of pristine lignin (SEM images) and (**c**) on the left: dispersion of lignin nanoparticles in PBS buffer solution (pH 7.4), on the right: microscopic morphology of lignin nanoparticles (SEM images).

To characterize the chemical structure of lignin extracted from coffee grounds, 2D-HSQC NMR analysis was performed (Figure 1a). The aliphatic regions exhibit prominent signals corresponding to *β-O-*4 (A), *β-β* (B), and *β-*5 (C) linkages. This observation indicates that the primary natural structure of lignin has been preserved. Additionally, signals corresponding to other aromatic components, such as guaiacyl (G), syringyl (S), and p-hydroxyphenyl (H) units, were detected in the aromatic regions of the samples. This suggests that the analyzed samples possess the characteristic structure of Gramineae lignin [11,20].

The bottle on the left side of Figure 1b illustrates the presence of stratification in the sample, indicating poor dispersion. The scanning electron microscope (SEM) image (Jeol JSM-7800F) on the right side reveals that the particles are significantly agglomerated, with large and heterogeneous sizes, and micrometer-sized aggregated structures visible in certain areas. In contrast, the left bottle in Figure 1c displays the sample as a uniform dark liquid with good dispersion. The SEM image on the right side shows that the particles are regularly spherical, indicating that the morphology and dispersion of the lignin particles have been significantly improved following the specific treatment. Figure 1b,c demonstrates the effects of preparation conditions or treatments on the morphology and dispersion of lignin particles by comparing the macroscopic dispersion state and microscopic morphology of different samples [11]. This comparison provides a structural and morphological basis for the study of the preparation and properties of lignin-based materials.

**Figure 2:**
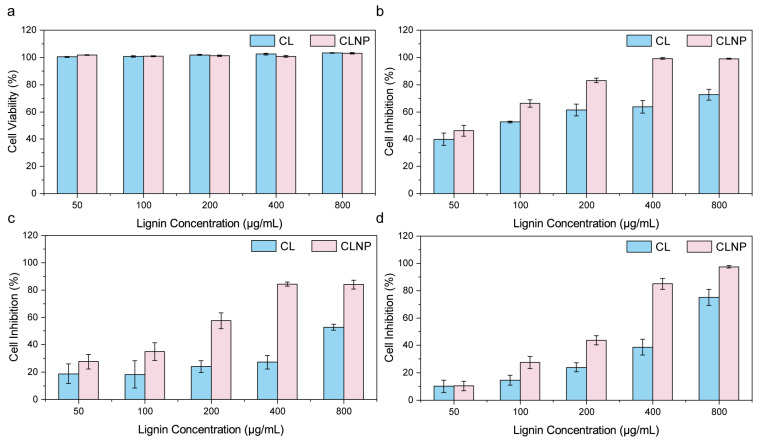
The cytotoxicity of coffee lignin (CL) and coffee lignin nanoparticles (CLN) on the human colon cell line (**a**) NCM460, as well as on CRC cell lines (**b**) SW620, (**c**) HT29, and (**d**) HCT116.

In vitro cytotoxicity of coffee lignin (CL) and its nanoparticle formulation (CLN) against three colorectal cancer (CRC) cell lines (SW620, HT29, HCT116) and one normal colon cell line (NCM460) was studied using the CCK-8 assay (Figure 2 and Table 1). In comparison with CL, CLN exhibited significantly stronger cytotoxicity towards all three CRC cell lines at all tested concentrations, while showing no obvious cytotoxicity against normal NCM460 cells. To be specific, CLN presented IC50 values 2.15–3.89 folds lower in comparison with CL, with the most prominent reduction observed in HT29 cells (from 778.54 μg/mL for CL to 199.74 μg/mL for CLN). The enhanced inhibitory effect of CLN on CRC cell lines can be attributed to several factors: (1) The nanoscale structure of lignin nanoparticles provides a larger specific surface area, significantly increasing the contact area with cancer cells. This enables more effective interaction with receptors or targets on the cancer cell surface, thereby enhancing the likelihood of an inhibitory effect [4]. (2) The nanoscale structure is more adept at penetrating biological barriers, such as the abnormal blood vessel walls of tumor tissues and cancer cell membranes. Additionally, cancer cells exhibit stronger endocytosis of nanoparticles, facilitating the entry of more lignin active ingredients into the cell interior, where they can directly target key components (e.g., cell nuclei, mitochondria) to inhibit proliferation or induce apoptosis [21]. (3) The surface charge, hydrophobicity, and other properties of lignin can be modified when prepared as nanospheres. For instance, an appropriate surface charge (e.g., positive charge) can create a stronger electrostatic attraction to the negatively charged cancer cell membranes, promoting adsorption and internalization. Furthermore, optimized hydrophobicity can improve solubility within the lipid bilayer of cell membranes, thereby facilitating transmembrane transport [11,20].

**Figure 3:**
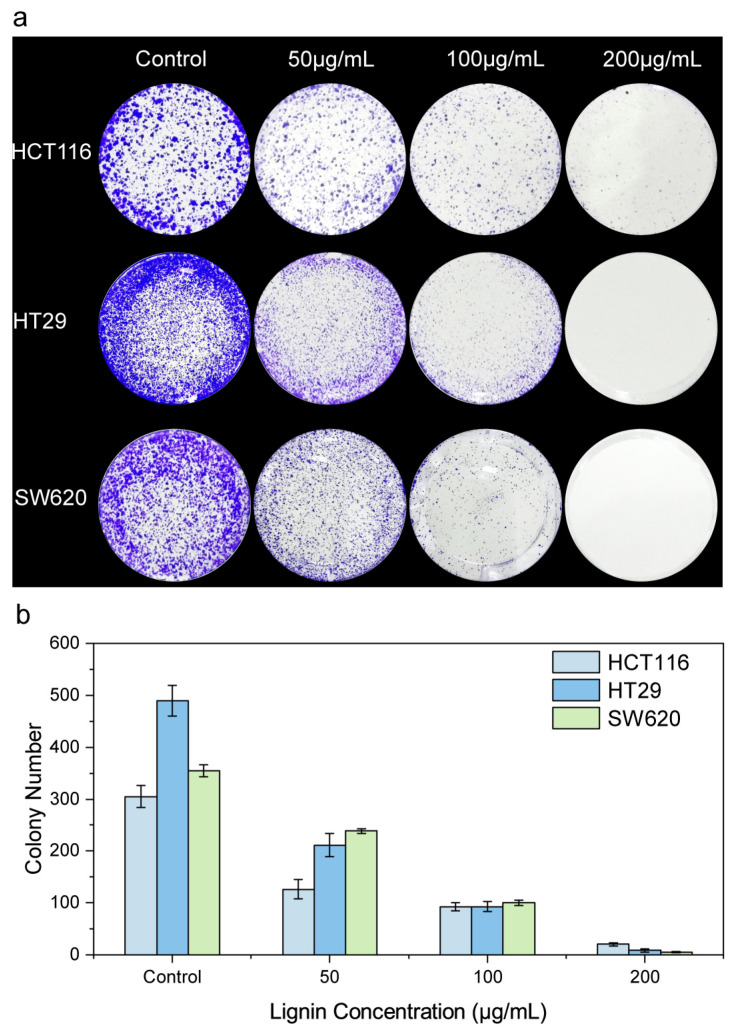
Effect of CLN on clone formation in three CRC cell lines (HCT116, HT29, SW620), divided into (**a**) microscopic images of clone formation and (**b**) data statistics of clone numbers.

To evaluate the effect of CLN on the clone formation ability of CRC cells, different concentrations of CLN were used to treat CRC cell lines, and the number of clones was quantified. Figure 3 illustrates the impact of CLN on clone formation in CRC cell lines (HCT116, HT29, and SW620). Figure 3a presents microscopic images of cell clone formation, with each row corresponding to a specific cell line. From left to right, the groups are arranged as follows: control (0 μg/mL), 50 μg/mL, 100 μg/mL and 200 μg/mL of CLN treatment. The blue spots in the images represent cell clones. It is evident that the number of clones gradually decreases as the concentration of CLN increases, indicating that CLN exerts an inhibitory effect on cell clone formation. Figure 3b (Tabel 2) illustrates the histogram statistics of clone numbers, in the control groups, HCT116, HT29, and SW620 cells exhibited clonogenic counts of 305.07 ± 20.92, 489.45 ± 29.64, and 354.6 ± 12.13, respectively. Following treatment with CLN, the clonogenic potential of all three cell lines was significantly attenuated. Notably, the most prominent inhibitory effect was observed at the maximum tested concentration of 200 μg/mL, with clonogenic counts reduced to 20.00 ± 2.70 (HCT116), 8.61 ± 3.36 (HT29), and 5.09 ± 1.53 (SW620), respectively. All of which were significantly different from the control group (*p* < 0.05). In summary, the control group exhibited a high number of clones for all three cell lines; however, as the concentration of CLN increased, the number of clones decreased significantly, reaching a very low level at 200 μg/mL. These results demonstrate that CLN exerts a concentration-dependent inhibitory effect on the formation of clones in the three CRC cell lines [22]. The higher the concentration, the more pronounced the inhibitory effect, highlighting the potential of CLN in suppressing the clone formation of CRC cells (Table 2).

**Figure 4:**
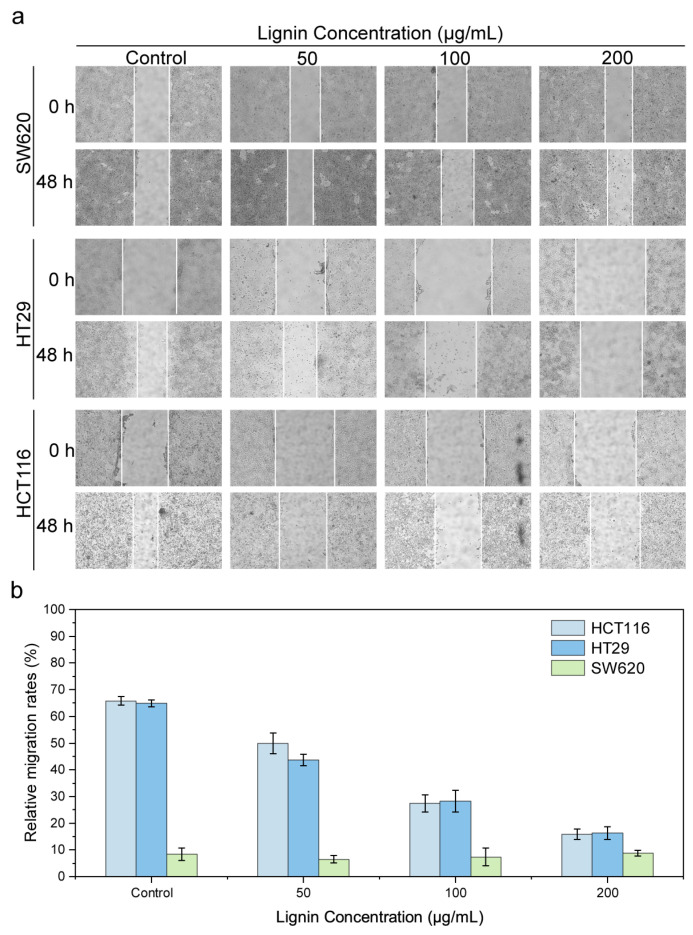
Effect of CLN on the migration ability in three CRC cell lines (HCT116, HT29, SW620), divided into (**a**) microscopic images of clone formation and (**b**) data statistics of clone numbers.

In the present study, the influence of coffee grounds-derived lignin nanoparticles (CLN) on the migratory potential of three colorectal cancer (CRC) cell lines (SW620, HT29, HCT116) was systematically assessed via cell scratch assay. As illustrated in Figure 4, the control group (0 μg/mL CLN) exhibited prominent scratch healing, whereas CLN treatment exerted a substantial concentration-dependent inhibitory effect on the migration of HCT116 and HT29 cells [23]. Notably, at the maximum tested CLN concentration (200 μg/mL), the 48 h migration rates of both cell lines were reduced to approximately 16%. However, no significant migration was observed in SW620 cells regardless of CLN treatment, which may be ascribed to the inherent biological characteristics of this cell line. Collectively, these findings demonstrate that CLN possesses considerable anti-migratory potential against HCT116 and HT29 cells, thereby holding promise for suppressing the invasion and metastasis of these CRC subtypes.

**Figure 5:**
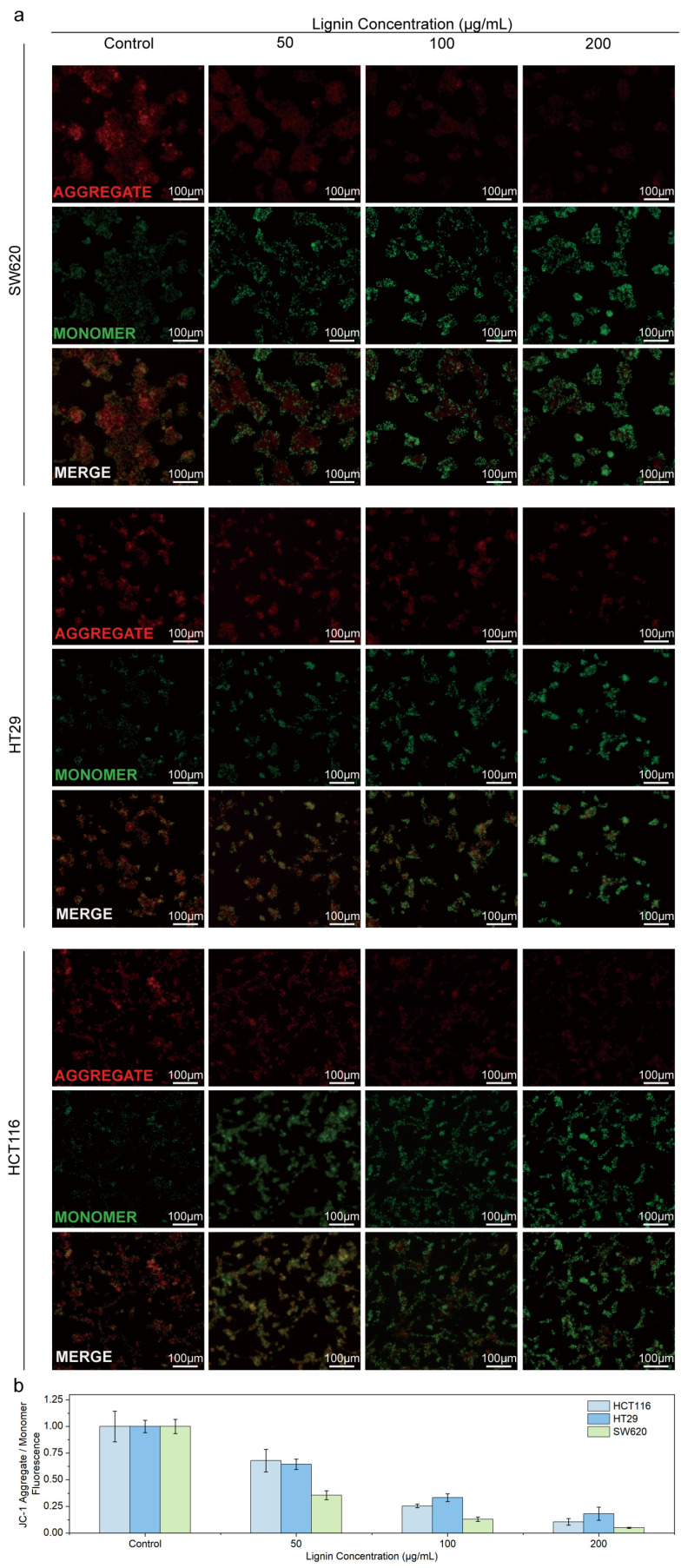
Lignin disrupts mitochondrial membrane potential (MMP) in cancer cells. (**a**) Representative fluorescence images of three CRC cell lines (HCT116, HT29, SW620) stained with the JC-1 dye after treatment with varying concentrations of lignin. A shift from red fluorescence (JC-1 aggregates, high MMP to green fluorescence (JC-1 monomers, low MMP) indicates mitochondrial depolarization. (**b**) Quantitative analysis of the JC-1 aggregate-to-monomer ratio in untreated CRC cells or CRC cells following treatment with a specific concentration of lignin.

To clarify whether CLN induces apoptosis in CRC cells by interfering with mitochondrial function, the present study investigated the effect of CLN on the mitochondrial membrane potential (MMP) of CRC cell lines (HCT116, HT29, SW620) via JC-1 staining combined with fluorescence imaging and quantitative analysis. Figure 5a presents fluorescence inverted microscopy images depicting the mitochondrial membrane potential of CRC cells treated with varying concentrations of CLN (0, 50, 100, and 200 μg/mL), with cells labeled using the JC-1 dye. The red fluorescence (aggregate) signifies the polymer formed at high MMP, while the green fluorescence (monomer) indicates low MMP. As the concentration of CLN increased (0, 50, 100, and 200 μg/mL), the red fluorescence gradually diminished, and the green fluorescence increased correspondingly. This trend suggests that CLN treatment led to a reduction in MMP in CRC cells lines. Figure 5b (Tabel 3) provides the statistical analysis corresponding to Figure 5a, with different colors representing various cell lines. In control groups, red-to-green fluorescence intensity ratios were high, but these ratios significantly decreased with increasing CLN concentrations, confirming MMP dissipation. Collectively, these findings indicate that CLN disrupts the MMP of CRC cells, a change typically associated with the process of apoptosis [23]. CLN induces the release cytochrome C and other pro-apoptotic factors by affecting mitochondrial function, activating the caspase cascade reaction [24], and ultimately inducing apoptosis in CRC cells [25]. This effect specifically targets the reliance of cancer cells on energy metabolism and survival, CLN effectively inhibiting tumor growth [23]. It offers a novel target and strategy for the treatment of CRC, highlighting the advantages and potential of CLN in combating CRC by interfering with mitochondrial function (Table 3).

## 4. Conclusions

In this study, we systematically evaluated the potential of coffee grounds lignin (CL) and its nanoparticles (CLN) in the treatment of CRC using 2D-HSQC NMR, SEM, and other characterization techniques, as well as cellular-level experiments. At the structural level, lignin retains its natural β-O-4, β-β linkages and other characteristic structures, along with guaiacyl/syringyl/p-hydroxyphenyl (G/S/H) units, which provide the material basis for its bioactivity. Through nanosizing treatment, the dispersion and targeting of CLN are significantly enhanced. Its approximately 100 nm homogeneous globular structure not only increases the interaction area with tumor cells but also overcomes the traditional limitations of lignin’s poor solubility, establishing a morphological foundation for efficient delivery. The cytotoxicity and functionality experiments demonstrated that CLN exhibited no significant toxicity to normal colon cells (NCM460) while effectively inhibiting the proliferation of CRC cells, such as HCT116, HT29 and SW620, in a concentration-dependent manner. Notably, the inhibition rate of CLN at 400 and 800 μg/mL was significantly higher than that of untreated lignin (CL), underscoring the efficacy of nanosized lignin. Mechanistic studies further revealed that CLN activated the apoptotic pathway in colorectal cancer cells by reducing mitochondrial membrane potential, and inhibited their clone formation and migration, preliminarily demonstrating the ability to obstruct colorectal cancer cell development. This dual inhibition of tumor energy metabolism and invasive capabilities highlights the distinct mode of action of lignin derivatives compared to traditional chemotherapeutic agents. From the perspective of application value, the extraction of lignin from coffee grounds waste, combined with the nanonization process, embodies both green chemistry principles and cost advantages. This approach presents an innovative paradigm of “high-value utilization of waste—precise targeted therapy” for the development of natural product anticancer drugs.

This study focuses on the in vitro inhibitory effects of coffee grounds-derived lignin nanoformulations (CLN) on colorectal cancer (CRC) cells, and only obtains preliminary data related to cytotoxicity and cell growth. Nevertheless, this study has clarified directions for the subsequent development of CLN in areas such as basic mechanism validation, formulation optimization, and safety evaluation. Future studies will not only verify the potential value of CLN in CRC treatment through in vivo experiments, but also improve the analysis of its action pathways via mechanistic assays to conduct in-depth investigations into the anticancer mechanism of CLN; additionally, emphasis should be placed on the in vivo pharmacokinetics of CLN, the development of targeted delivery systems, and the design of combination therapy strategies—all of which aim to facilitate the translation of laboratory findings into clinical applications. In summary, coffee grounds-derived lignin (CL) and its nano-formulations (CLN) exhibit potential scientific value in in vitro anti-colorectal cancer cell research, providing a new direction for the high-value utilization of coffee grounds and the development of natural product-based candidates against colorectal cancer cells. The research findings offer a replicable paradigm for the interdisciplinary integration of “waste—functional components—precision medicine”, spanning materials science, green chemistry, and oncology.

## Figures and Tables

**Table 1:** The cytotoxicity (IC_50_ value, μg/mL) of coffee lignin (CL) and coffee lignin nanoparticles (CLN) on CRC cell lines (SW620, HT29, and HCT116).

Cell Line	CL	CLN
SW620	85.68	33.11
Activity increase ^a^		2.58
HT29	778.54	199.74
Activity increase		3.89
HCT116	519.38	241.27
Activity increase		2.15

^a^ In comparison with CL.

**Table 2:** Quantitative analysis of the effect of CLN (μg/mL) on the clonogenic potential of CRC cells.

Cell Line	0	50	100	200
HCT116	305.07 ± 20.92	126.42 ± 18.27	92.5 ± 7.87	20.00 ± 2.70
HT29	489.45 ± 29.64	211.34 ± 22.01	92.59 ± 9.66	8.61 ± 3.36
SW620	354.6 ± 12.13	238.76 ± 4.56	99.88 ± 4.90	5.09 ± 1.53

**Table 3:** Migration rates of CRC cells under CLN (μg/mL) treatment.

Cell Line	0	50	100	200
HCT116	65.81 ± 1.61	49.88 ± 3.83	27.34 ± 3.23	15.82 ± 1.94
HT29	64.9 ± 1.23	43.77 ± 2.14	28.26 ± 4.09	16.33 ± 2.36
SW620	8.36 ± 2.33	6.52 ± 1.47	7.34 ± 3.29	8.78 ± 1.17

## Data Availability

Data are contained within the article.
